# Senior Nurse Manager Perceptions of Nurse Practitioner Integration: A Quantitative Study

**DOI:** 10.1155/2024/9956994

**Published:** 2024-06-17

**Authors:** M. Ryder, G. Lowe, P. Gallagher, V. Plummer, J. Mcentee, A. Driscoll, E. Furlong

**Affiliations:** ^1^School of Nursing, Midwifery and Health Systems, University College, Dublin, Ireland; ^2^Institute of Health & Wellbeing, Federation University, Ballarat, Australia; ^3^Nursing & Midwifery, Ireland East Hospital Group, Dublin, Ireland; ^4^Nursing & Midwifery, RCSI Hospital Group, Dublin, Ireland; ^5^School of Nursing & Midwifery, Deakin University, Centre for Quality & Patient Safety Research (QPS), Austin Hospital, Geelong, Australia

## Abstract

**Aim:**

To determine Senior Nurse Managers' perceptions of integration of Nurse Practitioner roles in Healthcare Organisations across Ireland and Australia.

**Background:**

Introduction of the Nurse Practitioner role in both countries is well established with national policies aimed at developing a critical mass in the health workforce. Current policy requires Senior Nurse Managers to be actively involved in the introduction of and oversight of the integration of Nurse Practitioners across healthcare settings. This is integral in the context of the success and sustainability of the services provided by the Nurse Practitioner.

**Methods:**

A quantitative, cross-sectional cloud-based survey of senior nurse managers across Ireland and Australia from April to September 2022.

**Results:**

Of 300 responses received, 122 were eligible for analysis. Of these, 77% expressed that there should be a specific role to support the integration of Nurse Practitioner roles at local level, and 61% recommended that this should occur at a national level, whilst 48% reported the absence of a standardised governance structure. Three reporting structures were identified: professional, clinical, and operational. Autonomous clinical decision making and prescribing were two Nurse Practitioner functions most identified. Fifty-five percent reported having performance indicators for Nurse Practitioner roles, with 24% agreeing that performance indicators captured the quality of care provided. Thirty-five percent of senior nurse managers indicated that there were agreed reporting timelines for performance indicators and a requirement for the provision of an annual report.

**Conclusion:**

Whilst some participants reported structure to guide and evaluate the work and value of Nurse Practitioners, the approach was inconsistent across organisations and countries. This paper demonstrates that integration is not broadly established across both countries. *Implications for the Profession*. The main findings were that Nurse Practitioners were misunderstood and the development of a structured framework to support the integration of Nurse Practitioners would provide long-term benefits.

## 1. Introduction

Nurse Practitioners (NPs) are clinical nurse leaders and a key component in healthcare transformation and healthcare reform [1]. In response to the current global workforce challenges and increased demands on health services, NPs have been shown to reduce fragmentation of care and improve the patient journey whilst improving efficiency of service provision [2] and to be cost-effective [3]. NPs practice autonomously and independently within agreed caseloads in the Irish context and scopes of practice in the Australian context [4]. They undertake care across broad contexts and settings—wide-ranging both geographically and clinically—and are capable of reducing health service gaps and increase services where wait times for treatment are excessive [5, 6]. Further, evidence supports a reduction in hospital transfers when NPs are involved in care [7], that hospital readmissions reduced and safe prescribing increased with the inclusion of NPs in service delivery [6, 8].

Integration of NPs for the purposes of this research is referred to as a process of embedding the roles into the healthcare teams through organisation change at the micro and macrolevels [9]. The integration of NP roles depends on a structured, multilevel support system to ensure they are embedded within the service rather than remain as an “add-on” [2, 10]. The complex multilevel support required involves clinical support to facilitate clinical practice, team support whilst team dynamics are being redesigned, system-wide support in the form of strategic direction from nurse managers, and financial support from the organisation [10]. This framework by Contandriopoulos et al. [10] was previously used to explore NP perceptions of integration in the Irish context [11].

Previous research has identified that the sustainability of NP roles requires planning and support to ensure the role is adequately integrated into service provision [2]. Despite this, recent research identified that a structured approach was lacking in relation to the integration of NP roles into acute healthcare organisations, potentially leading to missed opportunities to demonstrate the true value of NPs in healthcare and long-term sustainability of roles [11]. These factors support further research in the area on NP integration.

Whilst there are a number of NP integration frameworks emerging, there is no international collaboration to date on this issue. Previous research has affirmed that the NP regulatory framework and roles are comparable [4]. The research team concluded this was an opportunity to explore NP integration from an international perspective between countries with similar regulatory frameworks.

## 2. Background

Previous work has highlighted the need for effective leadership to drive changes necessary for NP integration and to overcome the barriers presented by existing organisational structures and professional cultures [2]. Leadership, specifically through challenging current structures and facilitating and enabling change of healthcare service delivery, is supportive of NP integration [2]. Integration of NP roles is reported as problematic globally, with inconsistent utilisation of NPs, and ineffective policy and governance issues [12, 13]. Porat-Dahlerbruch et al. [13] identified macrolevel NP integration factors at national policy level, such as pay scales and focused healthcare policies related to healthcare delivery. Organisational factors and institutional policies supporting streamlined governance structures are known as mesolevel factors, and microlevel factors relate to inter and intraprofessional relationships and clear understanding of roles and responsibilities within healthcare teams [13]. Chouinard et al. [14] identified that Directors of Nursing were the key stakeholders enabling NPs to enact their role to its full potential and that nurse managers are the most useful source of support to define and develop NP roles in a primary healthcare setting.

Current Department of Health policy in Ireland supports the increase of Registered Advanced Nurse Practitioner (RANP) roles to achieve a critical mass target of 2% of the workforce [15] in targeted areas. This target has been achieved (Nursing and Midwifery Board of Ireland [16]). An evaluation of this policy implementation in Ireland by Brady et al. [6] referred to the key barrier to successful implementation of the Department of Health policy was the requirement for organisational governance. There is no standardised governance structure for NP integration in Ireland [11].

In the current Australian context, the Nurse Practitioner Workforce Plan (called The Plan) aims to increase the number of NP roles nationally and ensure they are working to their full scope of practice [15]. The Plan [15] acknowledges that the integration of NPs into workforce planning assists in a whole of workforce planning framework, achieved through better understanding of roles by health workforce planners, healthcare teams, and consumers of health.

Despite the varying national policies, previous research comparing the NP roles across Ireland and Australia has determined that there is no significant difference in the work of both roles [17]. The same research reported that the leadership activities were validated by NPs in both Ireland and Australia, supporting the similarities in both roles [4, 17].

A NP integration framework was proposed by Contandriopoulos et al. [10]. The framework identified five factors for the successful integration of NPs in a primary care setting. The original factors included planning, role definition, practice model, collaboration, and team support. The framework was previously modified to explore NP perceptions of integration in one healthcare region in Ireland. The modification resulted in the identification of four distinct categories in a NP integration framework which included planning, governance and support, role definition and consensus, collaboration and referral, and outcome and performance measurement. This modification is reported in detail by Ryder and Gallagher [11].

## 3. The Study

### 3.1. Aim

The study's aim was to determine senior nurse managers' perceptions about the integration of Nurse Practitioner roles in Healthcare Organisations across Ireland and Australia.

### 3.2. Objectives

The research questions were generated under four distinct categories for NP integration, defined by Ryder and Gallagher [11] The survey questions were rephrased from Ryder and Gallagher [11] for the purpose of this study: (1) planning, governance, and support; (2) role definition and consensus; (3) collaboration and referral; and (4) outcome and performance measurement. The objectives of this research are to

1. Identify the perceptions of governance and support necessary by nurse managers to integrate NP roles
2. Ascertain senior nurse managers' perceptions of role definition and whether consensus exists
3. Determine senior nurse manager perceptions of specific outcome and/or performance measures used to evaluate NP practice

## 4. Methods

### 4.1. Design

The study was a quantitative, cross-sectional cloud-based survey. Participants of interest were senior nurse managers across Ireland and Australia.

### 4.2. Setting, Sampling, and Recruitment

The population for the current study included senior nurse managers in Australia and Ireland. Primary recruitment was via social media and targeting professional nurse management associations in Ireland and Australia. Convenience and snowball sampling was used to enable participants to share the study information with colleagues and others within their organisations and networks. It is not possible to know the exact figures of the population as there was no database to describe this figure.

A snowballing technique was used to disseminate (1) a flyer and (2) a plain language information sheet (PLIS) providing details of the study aims, which also included a link or QR code to enable direct access to the online survey. The research team included managers, clinicians, and academics. The team disseminated the invitation to the survey among their own networks with a request to forward to other potentially suitable participants. The invitation included the flyer and/or the PLIS so the link could be accessed directly. The survey remained open online between June and September 2022. For the purposes of this study, senior nurse manager roles include

1. Director of Nursing
2. Assistant Director of Nursing
3. Directorate Nurse Manager
4. Director of Midwifery
5. Clinical Manager
6. Director of Education
7. NP/RANP (Australian and Irish terminology, respectively)

### 4.3. Sample Size

Convenience and snowball sampling was used to identify and recruit Senior Nurse Managers across Ireland and Australia. A power analysis was not undertaken for this study as it is not possible to determine the number of senior nurse managers working in Ireland or Australia.

### 4.4. Instrument

An online survey instrument was designed by the research team to survey senior nurse managers. The concept for the instrument was based on previous research by Lowe et al. [2]. The instrument was modified following an exploration of existing literature on the topic from Contandriopoulos et al. [10] who developed a framework for nurse practitioner integration in primary care. Development of the online survey instrument modified from Contandriopoulos et al. [10] framework is described in detail by Ryder and Gallagher [11]. There are four sections in the instrument of Ryder and Gallagher [11]. Questions in the instrument were adapted for senior nurse managers for the purpose of this research using Qualtrics® software.

The final instrument for this study was composed of four sections, namely, planning/governance and support (9 items); role definition and consensus (9 items); collaboration and referral (2 items); and outcome/performance measurement (9 items). In addition, the questionnaire included demographic items (5), questions exploring previous experience working with NPs (6 items), and two open-ended questions. There were a total of 42 items in the final survey instrument (available upon request). The survey was tested for face validity among five senior nurse managers working in healthcare regions in Ireland and Australia. The senior nurse managers were randomly selected by members of the research team. No modifications to the survey were recommended following review. Reliability was tested during analysis. The survey was hosted on the Qualtrics platform and accessed via a direct link or QR code.

### 4.5. Quality Appraisal

The first appraisal sought to identify incomplete responses. All responses with less than 70% completion were removed to ensure the quality of data collected was optimal [18]. Secondly, where responses indicated the role title was inconsistent with our inclusions (see Section 4.3) the responses were removed.

### 4.6. Data Abstraction

Data were exported directly from the cloud-based survey platform Qualtrics® to SPSS® for analysis.

### 4.7. Data Analysis

Data collected from the closed questions were analysed using IBM SPSS Statistics (Version 27). Descriptive statistics were used to summarise and describe the data. Content analysis was used for the open questions to determine themes, concepts, and relationships in the data, using a technique described in [19]. This process involved descriptions and interpretations of various levels of abstraction and interpretation [20].

### 4.8. Ethical Considerations

Submissions were made to the relevant ethics committees with approval numbers LS-E-22-23-Ryder and B22-022 provided as evidence of approval for the study. A participant information document was embedded into the survey which required selection of “I consent to participate” to access the cloud-based questionnaire. A flyer was prepared for social media recruitment with survey access links embedded. All responses were anonymous.

## 5. Results

A total of 300 responses were recorded in the cloud platform. Of those, 122 responses were identified as suitable for analysis. The results will be presented using subheadings consistent with those in the survey.

### 5.1. Demographics

The majority (*n* = 91; 75%) were in the 45–64-year-old age category. The mean years in nursing was 28 (*SD* 8) and mean years in the current role was 10 (SD 7). The majority were from Ireland (*n* = 106; 87%) and identified working in a metropolitan area (*n* = 52; 43%), Table 1.

All participants reported having previously worked with NPs or NPs in preparation, and 95% (*n* = 116) currently have NPs or NPs in preparation working in their organisation. The average number of NPs (whole time equivalent (WTE)) per organisation was 10 (SD 13; range 0–92) NPs in preparation *M* = 6; SD = 8; range 0–36.

Participants were asked to identify the top 3 gaps in service delivery that could be addressed by NPs to improve patient care. The most common areas identified were chronic disease management (*n* = 12), cardiology (*n* = 11), and mental health (*n* = 10), followed by diabetes and cancer (*n* = 8) and then respiratory and outpatient services (*n* = 6). Other clinical areas included dermatology (*n* = 5), women's health (*n* = 4), endocrinology and endoscopy (*n* = 2), and wound care (*n* = 1).

### 5.2. Planning, Governance, and Support

The second category in the survey related to items that identified opportunities for NP roles, support for integration into organisations, and advocates for the role. A majority expressed an opinion that there should be one specific role (single person) to fulfil this remit within an organisation (*n* = 94; 77%) and a single person responsible for identifying opportunities nationally (*n* = 75; 61%). The open text response indicated that this should be a senior nurse manager position at the level of Assistant Director of Nursing or above.

**Figure 1:**
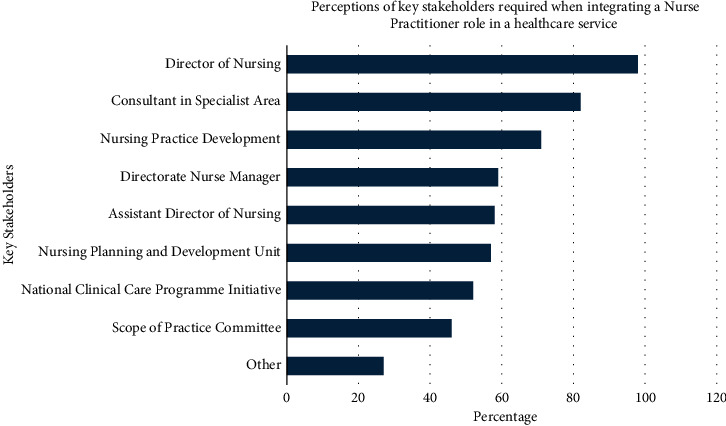
Key stakeholders required in healthcare organisation to integrate NP role. ^*∗*^Other: nurse practitioners/health service managers/patient representatives/administrative and financial personnel/departmental staff.

Participants identified the Director of Nursing position and the consultant (medical) in the specialist area as the two key stakeholders required when integrating the NP role into a healthcare service (Figure 1).

A higher proportion of participants (*n* = 59; 48%) reported that there was no standardised governance structure for the development/expansion and integration of the NP role in organisations. These participants selected that a standardised governance structure was required. The key stakeholders identified by participants for inclusion were senior nurse manager roles, consultants, allied health professionals, hospital governance, and service user representation to ensure roles are properly integrated into healthcare organisations.

Ninety-three percent of participants (*n* = 113) indicated a requirement for a three-dimensional reporting structure for NPs. This included professional reporting to the Director of Nursing (*n* = 95; 78%), clinical reporting to the medical consultant in specialist area (*n* = 89; 73%), and operational reporting with less clear responses to a Directorate Nurse Manager/Assistant Director of Nursing/Business Manager for the specialist area (*n* = 59; 48%). Additional reporting structures included regional and national nurse leaders and the regulator.

### 5.3. Role Definition and Consensus

**Figure 2:**
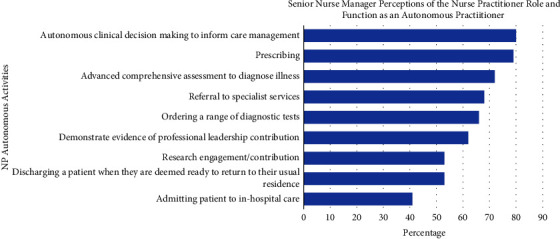
Senior nurse manager perception of NP autonomous functions.

Participants were asked to identify the autonomous functions of NPs from a list provided. The two functions identified most commonly were (1) autonomous clinical decision making to inform care management and (2) prescribing (Figure 2). The open text comments were related to (1) a lack of understanding by colleagues and other healthcare professionals of the NP role, (2) the need for additional time and support for NPs to engage with research outputs related to their service, and (3) concerns related to admission rights and referral for investigative procedures.

**Figure 3:**
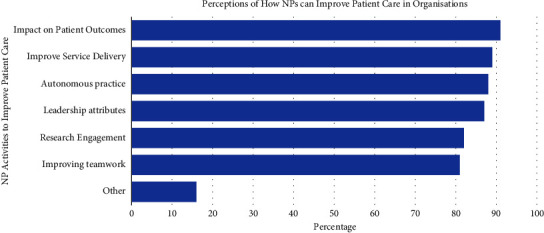
Senior nurse manager perceptions of how NPs can improve patient care in organisations. ^*∗*^Other: Continuity of care/educating staff/integration across acute and community/role modelling.

Participants were also asked to identify how they believed NPs could improve patient care within their organisation (Figure 3).

The majority (*n* = 102; 84%) indicated the presence of a specific job description for NPs within their organisation. Preparation of the job description was the role of the consultant in the relevant specialist area (*n* = 103; 84%) and the Director of Nursing (*n* = 98; 80%). Sixty-five participants (53%) reported that there was no agreed process to review NP roles and job descriptions (*n* = 53; 43%).

### 5.4. Outcome and Performance Measurement

Fifty-five participants (43%) reported that NPs in their organisation had agreed performance indicators (KPI). It was the opinion of some participants (*n* = 58; 47.5%) that the agreed KPI did not capture the quality of care provided by NPs. Forty-three participants (35%) reported that there were agreed KPI reporting timelines, with the same number (35%) reporting that NPs provide annual reports of activities. One question was related to the sustainability of NP roles. Participants were provided with a drop down option box and the ability to choose “all that apply.” The top three factors identified by senior nurse managers were (1) support from nursing management (*n* = 87), (2) organisational culture (*n* = 86), and (3) medical management support (*n* = 80).

Participants were provided with an opportunity to make any further comments to the researchers related to NP role integration through open text comment options in each category. Content analysis on the responses concluded that (1) work is required on the development of governance procedures to safeguard the future sustainability of the roles, for example, “annual report should be mandatory” and “national job description for specialties”; (2) integrating the role with interprofessional colleagues, for example, “a multidisciplinary committee so that stakeholders are engaged from the outset” and “multidisciplinary forums to present achievements”; and (3) that NP voices must be heard, for example, “NP/ANPs need to be more proactive and find a voice” and “NP/ANPs need a national voice.”

## 6. Discussion

There was a paucity of literature exploring senior nurse manager perceptions of NP roles internationally and this was the first time they had an opportunity to report their understanding of the roles against clear components of NP practice. A number of key findings were evident from the results of this study. First, whilst there was overall support for NP roles, a lack of understanding of the autonomous function of the role and scope of practice was evident. Further, governance of NP roles once implemented was reported as ad hoc and evaluation was unclear. Third, there was inconsistency between reporting research as an activity to improve patient care and understanding of NPs' engagement with research.

Consistent with previous literature, the first key finding of this study was that senior nurse managers in Australia and Ireland perceived that NPs were capable of autonomous practice and that their roles could have a positive impact on patient outcomes. Senior nurse managers reported a range of areas within their organisations where patient outcomes could improve with the addition of NP roles, particularly chronic disease management, cardiology, and mental health—areas identified as priorities by governments in both countries.

Whilst the majority of senior nurse managers (80%) reported their perceived understanding of the autonomous function of NPs included prescribing medicine and treatment, the majority of participants did not perceive that the autonomous role included admitting and discharging patients by NPs (41% and 52%, respectively). Despite national standards outlining admission and discharge of patients within the scope of NP roles [16, 21, 22], there is a discrepancy between the reported perception of these autonomous functions indicating a lack of understanding of these clinical responsibilities. This finding suggests that the role and function of NPs lacks clarity for some senior nurse managers and could result in missed opportunities for working to full scope of practice. The International Council of Nurses (ICN) definition of NP scope of practice is:

A Nurse Practitioner is an Advanced Practice Nurse who integrates clinical skills associated with nursing and medicine in order to assess, diagnose, and manage patients in primary healthcare (PHC) settings and acute care populations as well as ongoing care for populations with chronic illness [23].

This finding was consistent with recent evidence that NPs report a lack of understanding of their role across a variety of healthcare settings [4, 24]. It is also widely acknowledged by NPs themselves that their role is largely misunderstood, despite role clarity being essential to successful integration [25, 26]. With senior nurse managers identified as playing an important role in NP role integration [14], it is crucial that a full understanding of the current roles and future potential can be harnessed. The process of introducing and incorporating the NP role into any healthcare organisation is a key integration attribute to the successful outcome and sustainability of the role in the long term [13].

The second key finding was that senior nurse managers indicated very high expectations of NPs' ability to improve service delivery and patient care outcomes. There was a clear indication that NPs improve healthcare service delivery within organisations, which was consistent with previous literature [1]. However, a concern identified in this research indicated that there was a lack of agreed process to review or evaluate the NP roles, the NP patient population/eligible patients, and job descriptions. Almost half (44%) of senior nurse managers reported that NPs have outcome/performance measures, yet they reported that (1) the performance measures did not accurately capture the quality of care (24%); and (2) that outcome/performance measures were not a priority for role sustainability (46%)—again possibly due to the lack of clarity and/or understanding. Whilst Chouinard et al. [14] reported that Directors of Nursing were the key stakeholders enabling NPs to enact their role to its full potential and that nursing managers are the most useful source of support to define and develop NP roles in a primary healthcare setting, managers seldom reported meeting with NPs to clarify their roles. There are several reasons why it is vital to evaluate all NP services. There are several reasons why it is vital to evaluate all NP services, including; 1) planning for future workforce, 2) understanding patient sensitive outcomes, 3) identify the effects on organisational goals and 4) demonstrate and showcase the outcomes associated with NP roles on patient care [27].

The third key finding was the incongruity in senior managers' perceptions of NP engagement in research activities. Most participants reported that engaging in research activities would improve patient care; however, understanding of this activity as a function of the NP role was rated much lower. This finding is consistent with previous literature, with results that (1) 3% of NP work related to research, (2) all NPs indicated it was not supported by nurse managers, and (3) most NPs completed research in their own time, suggesting a misunderstanding of what constitutes research engagement [4, 17]. A translational research framework for NPs has been proposed in the literature which identifies the range of NP activities aligned to research engagement within the framework [11]. Examples of the activities included are implementing evidence-based practice (EBP) through guideline development and evaluation of the NP services provided. Smigorowsky et al. [28] propose that little is known about the outcome of NP work due to the poor quality of research produced. Failure to understand the value and importance of research engagement and supported time dedicated to research activities within the role inhibits the development and enhancement of research skills.

### 6.1. Strengths and Limitations

One of the key strengths of this research is that it is the first time there have been clear variables provided for senior nurse managers to report their understanding of the NP roles.

Limitations of the research include factors associated with the response rate which made it difficult to stratify the results by role of employment. The sample population was consistent across both countries; however, there were more responses from Ireland than Australia and legislative differences may have impacted responses. The role of individual participants may also have influenced their responses.

### 6.2. Recommendations for Further Research

The first recommendation for further research is the development of a framework to support the structured approach to NP integration across health services. The framework should include the three multilevel factors described by Chouinard et al. [14] but further incorporate the horizontal layers necessary for NP clinician and nurse manager support across systems [14]. The second recommendation is for NP job descriptions to be established as live documents, regularly reviewed by the NP and senior nurse manager. This third recommendation is to support the accurate evaluation of the value NPs add to service and therefore patient outcomes including the value of research engagement.

## 7. Conclusion

The study has highlighted that there continues to be positive perceptions of the ability for NPs to improve service delivery and patient care. However, understanding of the full scope of practice of NPs and therefore the potential benefits to health services is yet to be identified and fully appreciated. This work is aimed at the mesolevel described by Porat-Dahlerbruch et al. [13] with findings supporting the need for a structured approach to NP integration in healthcare organisations [29].

### 7.1. Implications for the Profession

The main findings indicate that NPs were misunderstood and that the development of a structured framework to support the integration of Nurse Practitioners would provide long-term benefits.

## Figures and Tables

**Table 1:** Demographics.

	Ireland	Australia	Total
	*N* (%)	*N* (%)	*N* (%)
*Age*			
25–44 years old	29 (24)	5 (4)	34 (28)
45–64 years old	77 (63)	26 (21)	103 (84)

*NPs in organisation*			
Previous experience working with NP/candidate NP (NP in preparation)	96 (79)	26 (21)	122 (100)
Currently working with NP/candidate NP (NP in preparation)	90 (74)	26 (21)	116 (95)

Place of work ^*∗*^*(total* *=* *135)* 13 people selected more than one option			
Metropolitan hospital	52 (43)	14 (11)	66 (54)
Metropolitan community	7 (6)	1 (1)	8 (7)
Regional hospital	28 (23)	7 (6)	35 (29)
Regional community	3 (2)	1 (1)	4 (3)
Rural hospital/community	14 (11)	4 (3)	18 (14)
Remote area	2 (2)	2 (2)	4 (4)

^∗^Participants were permitted to select more than one option for work location.

## Data Availability

The survey data used to support the findings of this study are available from the corresponding author upon request.
